# Trilogy Development of Proopiomelanocortin Neurons From Embryonic to Adult Stages in the Mice Retina

**DOI:** 10.3389/fcell.2021.718851

**Published:** 2021-10-05

**Authors:** Xuhong Zhang, Xiaoyu Wang, Senjie Wang, Wei Peng, Rahim Ullah, Junfen Fu, Yudong Zhou, Ye Shen

**Affiliations:** ^1^Department of Ophthalmology, The First Affiliated Hospital of Zhejiang University, Hangzhou, China; ^2^Center for Brain Research and Brain-Machine Integration, School of Brain Science and Brain Medicine, Zhejiang University, Hangzhou, China; ^3^Department of Endocrinology, The Children’s Hospital, Zhejiang University School of Medicine, Hangzhou, China

**Keywords:** POMC-positive neurons, amacrine cells, development, retina, embryonic

## Abstract

Proopiomelanocortin-positive amacrine cells (POMC ACs) were first discovered in adult mouse retinas in 2010; however, the development of POMC-ACs has not been studied. We bred POMC-EGFP mice to label POMC-positive cells and investigated the development of POMC neurons from embryonic to adult stages. We found that POMC neuron development is mainly divided into three stages: the embryonic stage, the closed-eye stage, and the open-eye stage. Each stage has unique characteristics. In the embryonic stage, POMC neurons appeared in the retina at about E13. There was a cell number developmental peak at E15, followed by a steep decline at E16. POMC neurons showed a large soma and increased spine numbers at the closed-eye stage, and two dendritic sublaminas formed in the inner plexiform layer (IPL). The appearance and increased soma size and dendrite numbers did not occur continuously in space. We found that the soma number was asymmetric between the superior and inferior retinas according to the developmental topographic map. Density peaked in the superior retina, which existed persistently in the retinal ganglion cell layer (GCL), but disappeared from the inner nuclear layer (INL) at about P6. At the same time, the soma distribution in the INL was the most regular. At the open-eye stage, the development of POMC neurons was nearly stable only with only an increase in the IPL width, which increased the soma–dendrite distance.

## Introduction

The retina is a superb model for analyzing the circuitry development, structures, and functions of the nervous system. There is a similarity between the development of the retina and the cerebral cortex (Voinescu et al., 2009). The development of retinal cells is regulated by the temporal expression of different transcriptional factors (Yan et al., 2020); however, the exact mechanism is still poorly understood. Neuropeptides also play an important role in development, and amacrine cells (ACs) are the major neuropeptide-secreting cells (Bagnoli et al., 2003). Sixty-three different types of ACs have been discovered (Nguyen-Ba-Charvet and Rebsam, 2020), and with the development of technology, new types of ACs are being confirmed. The ACs regulate the development and diseases of the retina via cell–cell interactions or neurotransmitter secretions (Masland, 2012). Furthermore, a few ACs have even shown their regenerative potential in the retinas of adult mice (Chen et al., 2015). The developmental pattern always provides clues about the pathophysiology of a disease (Finlay, 2008) and, thus, suggests therapies; therefore, it is essential to investigate the development of ACs.

Based on the type of inhibitory neurotransmitter ACs express, ACs are classified into four groups, including GABAergic ACs (43 types), glycinergic ACs (13 types), dual ACs (3 types), and nGnG ACs (4 types). Likewise, based on the neuropeptides they express, GABAergic ACs have been further classified into well-studied dopaminergic or tyrosine hydroxylase (TH) ACs, vasoactive intestinal peptide (VIP) ACs, and acetylcholinergic or choline acetyltransferase (AChT) ACs, and the newly discovered and poorly studied Proopiomelanocortin-positive amacrine cells (POMC ACs) (Gallagher et al., 2010).

It has been reported that POMC-expressing cells exist in the anterior and intermediate lobes of the pituitary, arcuate nucleus (ARN), and nucleus of the solitary tract (NTS) in the rat brain (Gallagher et al., 2010). Retinal POMC-ACs and other POMC-expressing neurons have a common embryonic origin. POMC is a complex precursor for several peptide hormones. The POMC prohormone cleaves into melanocyte-stimulating hormones (MSHs), the adrenocorticotropic hormone (ACTH), and β-endorphin. Proopiomelanocortin-positive amacrine cells have been classified as GABAergic AChT-ACs, and they express β-endorphin (Castro and Morrison, 1997). α-MSH also exists in the anterior part of the optic tract, the retinal epithelium, and cone photoreceptors in Xenopus amphibians (Teshigawara et al., 2001). ACTH, MSH, and β-endorphin are collectively called melanocortins. Melanocortins act via melanocortin receptors. Among five melanocortin receptors (MC1R–MC5R), MC3R–MC5R are abundant in retinal interneurons and glial cells (Wong et al., 1997; Wikberg et al., 2000). The μ-OR is located on ganglion cell dendrites within the inner plexiform layer (IPL) (Brecha et al., 1995). The presence of MCRs in the retina suggests a role for POMC in the visual system. Melanocortins are involved in background adaptation (Teshigawara et al., 2001), the development and repair of neurons (van de Meent et al., 1997), stimulation of neurite outgrowth (Rosellirehfuss et al., 1993), and metabolism and reproduction (McClellan et al., 2008; Diano, 2011).

The function of POMC in retinal physiology has been studied in adult animal models (Rosellirehfuss et al., 1993; van de Meent et al., 1997; McClellan et al., 2008; Diano, 2011); however, the development of POMC neurons in the mouse retina has not been studied. This study used a POMC-EGFP transgenic mouse model and investigated POMC neuronal development in the retina.

## Materials and Methods

### Animals and Procedure

POMC-EGFP mice [C57BL/6J-Tg (POMC-EGFP) 1Low/J; stock No:009593] were purchased from the Jackson Laboratory, and they had more than 99% cellular colocalization of EGFP and POMC peptides (Cowley et al., 2001). Mice were placed under a 12-h light–12-h dark cycle at room temperature (26°C). Male and female mice were put together for breeding, and pregnant females were separated after confirming the presence of a vaginal plug. We collected embryos at embryonic day 10 (E10), E13, E14, E15, E16, and E17 (at least six mice for each) as well as postnatal mice on P0, P2, P4, P6, P8, P10, P12, P14, P21, P28, P35, and adults. Three to four mice were collected at each timepoint. The left eyes were used for retinal sectioning, while the right eyes were used for retinal whole mounts. Food and water were provided *ad libitum*. All mice were executed at about 2 p.m. All procedures were carried out following the National Institutes of Health Guidelines for the Care and Use of Laboratory Animals and were approved by the Tab of Animal Experimental Ethical Inspection of the First Affiliated Hospital, School of Medicine, Zhejiang University (Approval No. 2021001).

### Immunofluorescence Staining

At set timepoints, the mice were anesthetized with pentobarbitone (50 mg/kg intraperitoneal), and myocardial perfusion was performed. The eyeballs were removed and fixed in 4% (w/v) paraformaldehyde in phosphate-buffered saline (PBS) for 2 h at 4°C. All the procedures were done under normal room light. The anterior part of the ocular globe, including the cornea, iris, and lens, was dissected under a microscope. The optic cup was suspended in 30% (w/v) sucrose solution. After dehydration, the optic cups were positioned in embedding medium (Neg-50; Thermo Scientific, Waltham, MA, United States) and frozen. We collected 15-μm-thick cryosections and air dried the slides at room temperature. Then the cryosections were washed with PBS for 10 min, dried, and blocked with confining liquid [10% normal donkey serum (NDS), 1% bovine serum albumin (BSA), and 0.3% Triton X-100 in PBS] for 1 h at room temperature in a humidity chamber. The sections were then dried, and the primary antibodies were added to the slides for 1 h at room temperature. The slides were washed with PBS three times (5 min each time) and then incubated with Alexa Fluor 488 or 546-conjugated secondary antibodies for 1 h at room temperature. After washing in PBS and drying, DAPI (1:4,000; C1002; Beyotime, Shanghai, China) was used to label the nuclei. The sections were then rinsed three times with PBS and mounted with 60% glycerine under coverslips. For retina whole mounts, retina tissues did not need dehydration, and the staining procedure was the same.

The primary antibodies were anti-AP2 (1:20, 3B5, DSHB, TX, USA), anti-RBPMS (1:1,000, ab194213, Abcam, MA, United States), and anti-Iba-1 (1:500, 019-19741, Wako, Japan). The secondary antibodies were donkey anti-rabbit IgG (1:1,000, A-21206; Thermo Fisher Scientific, MA, United States) and donkey anti-mouse IgG (1:1,000, A-21206; Thermo Fisher Scientific).

### Fluorescent Imaging

Retina whole-mount images (× 20) and × 20 retina section images were acquired with a virtual digital slice scanning system VS120 (6 slice system) fluorescence microscope (VS120, Olympus, Japan). Retina whole-mount images [× 60 (oil)] were acquired with Nikon laser-scanning confocal microscope (N-STORM/A1R, Japan). Some × 20 retina section images were also taken with a confocal microscope (FV1000; Olympus, Japan).

### Image Analysis

For × 60 cell single-soma analysis, we used the surface function of Imaris 9.0.1 to mask and obtain the volume, position, and ellipticity data. For × 60 cell single-spine analysis, we used the same function and obtained the volume and position data. We also adopted the spot function to obtain the spine number data. For × 20 section analysis, quantitation of the somas was performed using ImageJ (Fiji) software. For × 20 retina whole-mount analysis, we selected 30–40 areas for all quadrants in each retina to determine the cell density. We used the Imaris spot function to get the position of each cell, then used R (x64 4.0.4, ggplot2 package) and R Studio to plot a pseudo color scatter density map. According to the density map, we selected a 500 × 500-μm area of the dense and spare part spots in each layer, then analyzed the nearest neighbor distances (NNDs), and drew the distribution histography with the python software (python 3.8 64-bit). We calculated the regularity index as the mean NND/SD (standard deviation) (Casini et al., 1995). Data were statistically analyzed with GraphPad Prism 8, and the mean ± SEM (standard error of the mean) was used. Panels were put into multipart figures with Adobe Illustrator CC 2018.

## Results

### Section View of Proopiomelanocortin-Positive Cell Development

**FIGURE 1:**
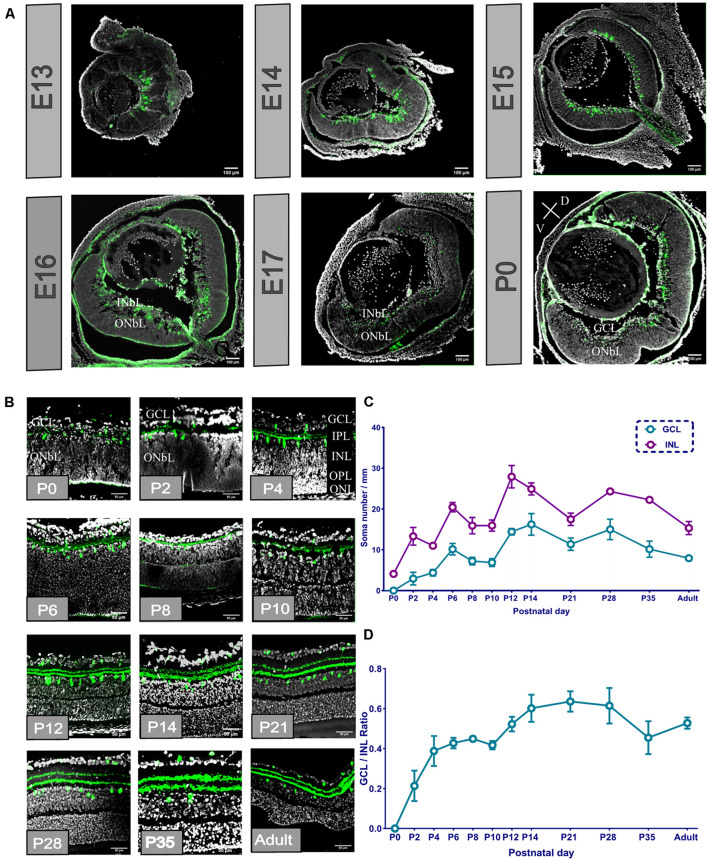
Proopiomelanocortin-positive amacrine cell (POMC AC) soma divided into layers, and the soma number and retinal ganglion cell layer (GCL)/inner nuclear layer (INL) ratio increased from the retinal section from E13 to adult. **(A)** Whole ocular section of E13–P0. POMC-positive cells (green) appeared at E13; the distribution changed from E16. Bar = 100 μm. Gray indicates DAPI. V indicates ventral retina, and D indicates dorsal retina. **(B)** P0-adult retina section. POMC-positive cell soma (green) divided into two layers at P2, and dendrites divided at P4 when the OPL appeared. Bar = 50 μm. Gray indicates DAPI. **(C)** POMC-positive cell soma number analysis in the two layers. The GCL number was less than the INL number at all time points. Both layer numbers increased from P0 to P14 and were stable to the adult stage (*n* = 12 for each timepoint). **(D)** GCL/INL number ratio. The ratio increased from P0 to P14 and kept stable to the adult stage at about 0.5 (*n* = 12 for each timepoint).

We dissected the whole eye of E13, E14, E15, E16, E17, and P0 mouse pups (corresponding to around E19), sectioned it, and selected the sections that had the optic disc and optic nerve (Figure 1A). From the E13 sections, we found that POMC-positive cells originated from the ocular posterior pole and covered approximately one-third of the retina. The cells were dispersed and ellipsoidal. At E14, axons appeared on the basal side of the retina, and cells covered about three-fourths of the retina. At E15, the area of the cells extended throughout the retina but still did not fully cover the retina. From E13 to E15, the retina had not been stratified. However, at E16, the retina developed into two layers, i.e., the loose inner neuroblastic layer (INbL) and the compact outer neuroblastic layer (ONbL). POMC-positive cells were dispersed in the INbL. The cell morphology became rounder, and the density was reduced. The basal side axons disappeared. At P0, the INbL developed into the retinal ganglion cell layer (GCL). There, the POMC-positive cell dendritic lamina moved toward the outer part within the GCL. POMC-positive cells were unevenly distributed along the two sides of the dendrite lamina, and the outer side had more cells.

From the enlarged section view (Figure 1B), the dendritic lamina at P0 was nearly single-lined, and the ONbL was not differentiated. At P2, the dendritic lamina was divided into two sublaminas, i.e., S1 and S4. Similarly, the POMC-positive cell somas also split into two layers, i.e., the INL and the GCL. At P4, the outer plexiform layer (OPL) appeared, and it divided the ONbL into two layers showing the outer nuclear layer (ONL). The distance between the two dendritic sublaminas increased. From P2 to P4, the cell soma numbers and the volume kept increasing and became more ellipsoidal. From P14 onward, the somas become more spherical. From P21, the POMC-positive cells were almost adult-like.

We analyzed the cell number of the two layers of POMC and the GCL/INL ratio (Figure 1C). The cell number showed three development peaks, i.e., at P2, P6, and P12. However, the ratio curve showed two peaks at P6 and P14 (Figure 1D). From P21 to adulthood, the cell number, distribution, and locations were almost stable, with a slightly decreasing trend in adults.

### Vertical View of Proopiomelanocortin-Positive Cell Development

**FIGURE 2:**
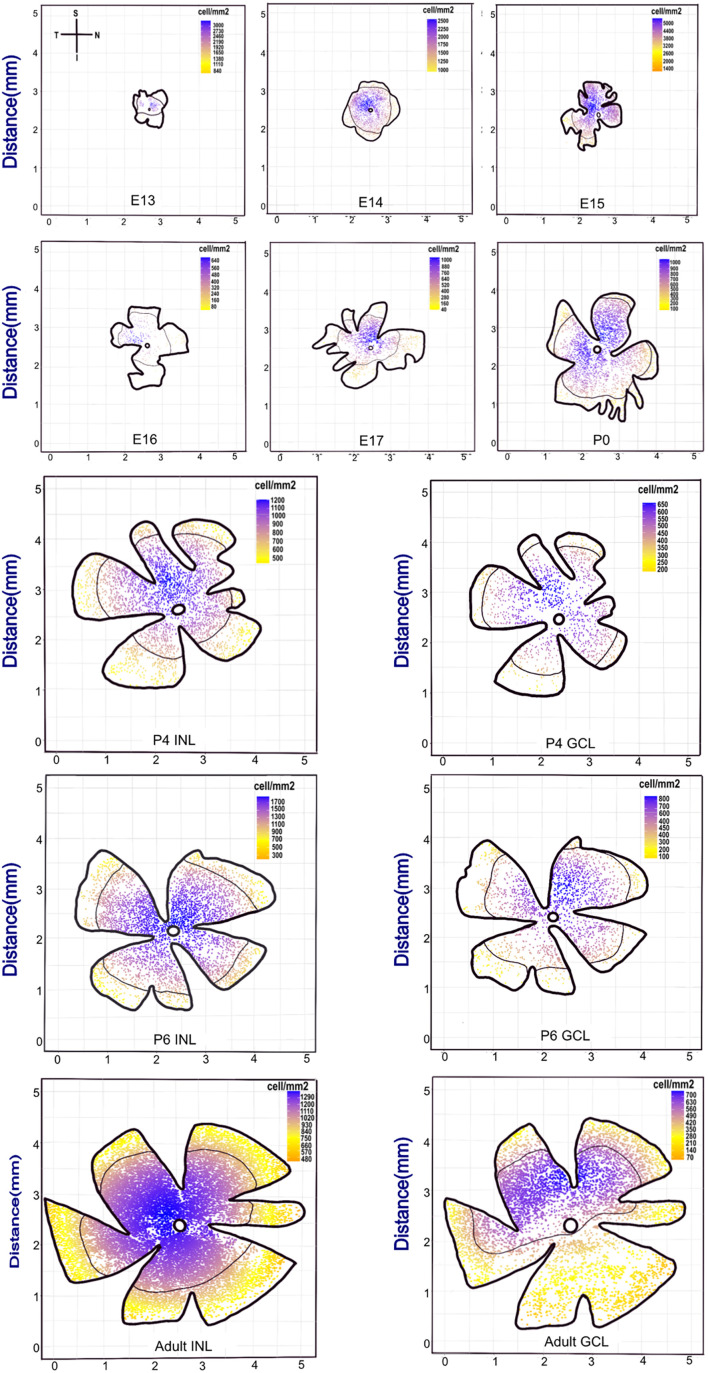
Retina whole-mount view showing that the POMC-positive cell distribution has a changeable dense pole. The coordinate axis indicates the actual retina size. From E13 to the adult stage, the retina whole-mount size became larger. Each retina was seated as superior to the upside and nasal at the right side. The scale bar represents the POMC-positive cell density for each retina. Blue represents the denser part, and yellow represents the sparse part. The black line shows the mean density contour line. E13 to P0 had only one layer, whereas P4, P6, and adult had two layers. The dense pole was always located in the superior retina from E13 to P6. However, from P6, the INL dense pole transferred to the optic disc, and the GCL pole was still in the superior retina.

From embryonic to adult stages, we found that the retina area extends from 1 to 25 mm^2^ (Figure 2 and Supplementary Figure 1). The distribution of POMC-positive cells was uneven at E13. The superior (dorsal) retina contained more cells, leading to an asymmetric distribution between the superior and inferior retina; however, it was nearly even at E15 and became uneven again at E16. From then on, cells in the INL gathered at the superior retina until P6 and then shifted to the optic disc, whereas the cells in the GCL were always gathered at the superior (dorsal) retina.

**FIGURE 3:**
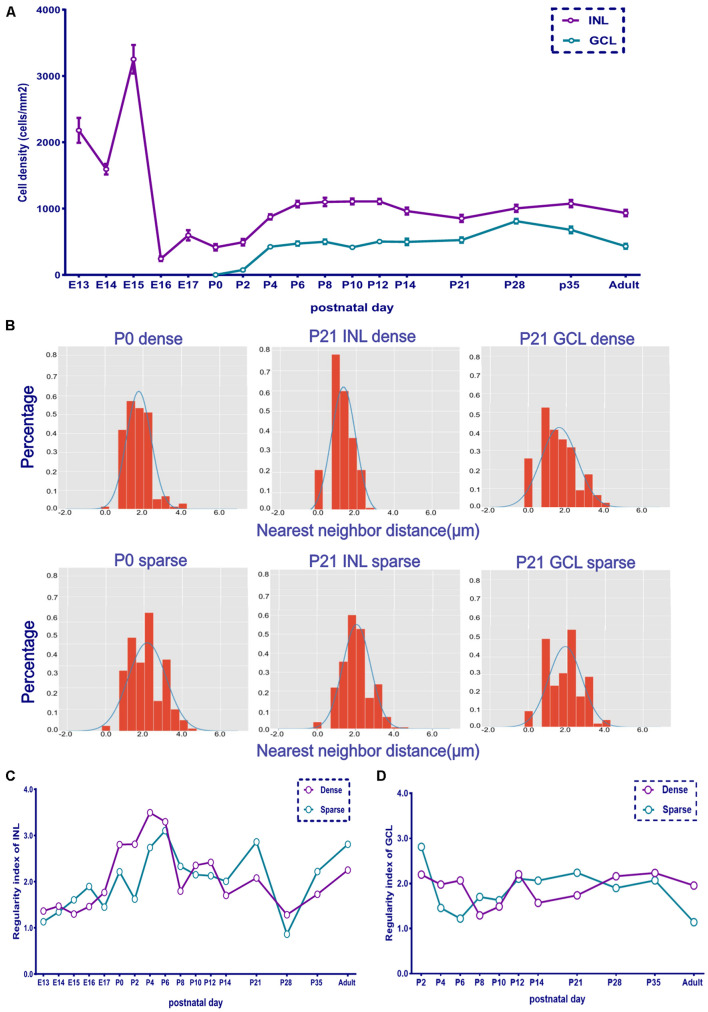
The retina whole-mount distribution of POMC-positive cells was changeable as indicated by the nearest neighbor distances (NND) and regularity. **(A)** Retina mean POMC-positive cell density of the two layers from E13 to adult. The INL was denser than the GCL, and the GCL/INL ratio was about 0.5 in adults (*n* = 12 for each timepoint). **(B)** NND frequency distribution histogram. The upside shows the dense area, and the downside shows the sparse area. Two images of P0 and four images of P21 are shown. The distribution was not random compared with the Gaussian distribution curve. **(C)** INL dense and sparse area regularity development curve (*n* = 3 for each timepoint). **(D)** GCL dense and sparse area regularity development curve (*n* = 3 for each timepoint).

The cell density of the INL showed considerable variation. It was 2,180 cells/mm^2^ at E13 and then decreased to 1,595 cells/mm^2^ at E14. It then increased and peaked at E15 (∼3,253 cells/mm^2^) and suddenly dropped at E16 (∼244 cells/mm^2^). Afterward, it increased gradually. The cell density of both layers showed a peak from P4 to P12 and then decreased to the adult stage (∼1,000 cells/mm^2^ for INL and 500 cells/mm^2^ for GCL; Figure 3A).

The dense and sparse part areas in both the INL and GCL showed a similar developmental trend of distribution regularity. The low regularity index at E13 suggested a random distribution. The regularity index increased and peaked at P4 (INL dense part) and P6 (INL sparse part) and then decreased again (Figure 3 and Supplementary Figure 2). The GCL showed a similar trend from P4 to P8; however, from P0 to P10, the GCL was less regular than the INL, while from P21 to adulthood, the GCL was more regular than the INL (Figures 3C,D).

**FIGURE 4:**
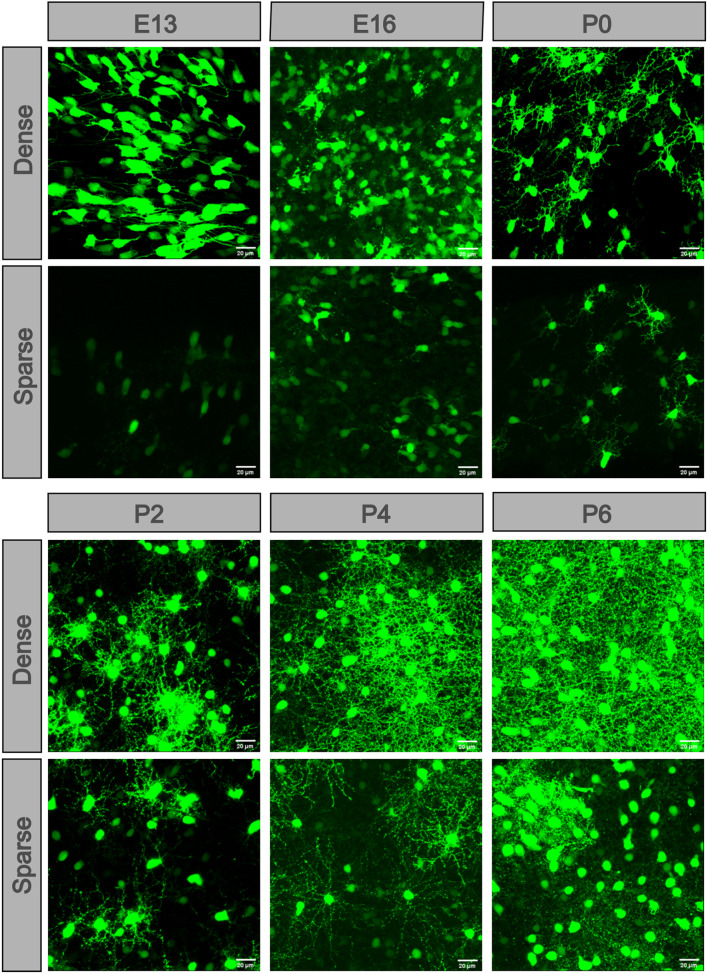
POMC-positive cells developed at different levels in different retinal areas. In each retina, cells located in the dense part were more developed. Dendrites appeared at P0 and then overlapped at P2. The more developed cells were separated by less developed cells. At P8, the dendrite net was throughout the full field. Bar = 20 μm.

To see the detailed difference between the dense and sparse part areas of the unevenly distributed retina at E13, E16, and P0–P2, we compared the dense and sparse part areas in the fluoroscopic images (Figure 4). At E13, there were numerous POMC-positive cells with axons extending in the same direction in the dense part. However, a few cells without axons were in the sparse part. At E16, the cell morphology showed the same pattern, but the number of cells increased. At P0, POMC-positive cells had both axons and dendrites. Dendrite fields of the adjacent cells overlapped and kept increasing from P0 in the dense part, whereas it was from P2 in the sparse part. Cell development with increased number and enriched dendrites was incontiguous in the sparse part.

**FIGURE 5:**
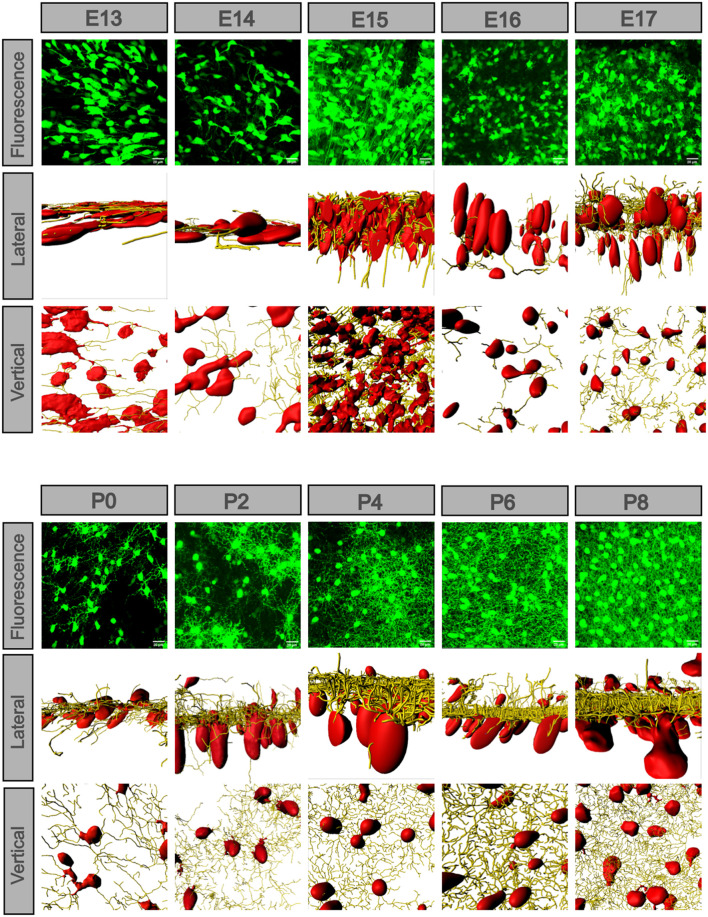
POMC-positive cell axons and dendrites exchanged along with the soma morphology. The fluorescence image was z-stacked. The vertical and lateral view images were masked by the Imaris surface and filament function. From E13 to P8, the lateral view showed that cell soma changed from flat to ellipsoidal and gradually separated into two layers, and so did the dendrites. At E13 and E14, there were only axons in the same direction; at E15, the axons were strongest. At E16, the axons disappeared, and dendrites developed. From P0 to P8, the dendrites formed two sublaminas and were regularly weaved. From a vertical view, the cell density changed, and dendrites gradually filled the whole field. Bar = 20 μm.

To see the developmental detail between the soma and dendrites, we selected the densest part of each retina from E13 to P8 (Figure 5), and at some timepoint, we used the same image from Figure 4 representing the dense part. We matched the cell soma with the surface function and the dendrites with the filament function using the Imaris software. The somas were oblate and sparse at E13, became thicker at E14, and were more ellipsoidal and dispersed at E15. From P0, the GCL showed somas, and they became larger and more ellipsoidal. The axons were fewer and plain at E13, and then became longer, stronger, and denser from E14. Dendrites were developed opposite the axons at E15 and became dense from then on. At E16, the long axons disappeared. The dendrite layer was not divided at P0, but at P2, there were two evident dendritic sublaminas, cross-linking irregularly. At P4 to P8, the dendrites were full field (vertical view), and the two sublaminas cross-linked more regularly (lateral view). However, spines appeared between the two sublaminas (enlarged section view of the IPL, Supplementary Figure 3).

**FIGURE 6:**
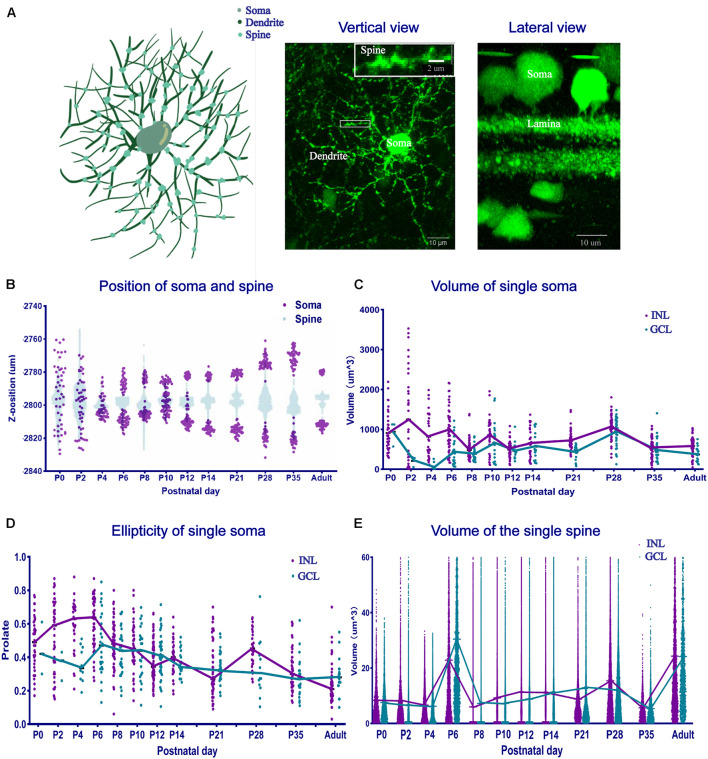
The position, volume, and morphology of single-cell soma and spines changed. **(A)** The schematic and fluorographs show the distinction of soma, dendrites, and spines. The white box shows an enlarged image area. Bar = 10 μm in the vertical and lateral view. Bar = 2 μm in the enlarged image. **(B)** Single-cell soma and spine position distribution. At P0 and P2, both the soma and spines were scattered and then gathered into two groups at P4. The distance between the soma layers became large. **(C)** Single-soma volume development. Single-soma volume showed increased uniformity. The INL volume increased, then decreased, and became stable at the adult level. The GCL volume decreased first, then increased, and became stable at the adult level. **(D)** Single-soma morphology development. The morphology distribution was always dispersive at different time points. **(E)** Single-spine volume development. The mean single-spine volume was nearly the same, with two peaks at P6 and in adulthood (*n* = 4 for each timepoint in all the panels).

We defined a POMC neuron as having a soma, dendrites, and spines, as shown in the schematic and fluorographs (Figure 6A). The soma position was decentralized at P0 and P2, gathered to the INL side at P4, and then the two groups of GCL and INL neurons were separated at P6, and the distance between the two groups increased with time (Figure 6B). The dendrite position also showed the same changes. From P12 onward, the thickness of S1 and S5 increased. The single soma volume changed from discrepant to uniform (Figure 6C). The soma volume of the INL reached the maximum value at P2 (1247.18 ± 33.88 μm^3^), then decreased from P2 to P8 (479.37 ± 4.12 μm^3^), and then stayed stable from P8 to the adult stage (∼500–700 μm^3^). In the GCL, it decreased from P0 to P4 (68.64 ± 26.37 μm^3^), then increased and stayed stable from P6 to the adult stage (469.94 ± 9.15 μm^3^). We noticed that, surprisingly, the soma volume in both layers was slightly increased (∼940.95–1,059.36 μm^3^) at P28. For the soma morphology (Figure 6D), the ellipticity in the INL was increased from P0 to P6 (0.60 ± 0.02), then decreased to the adult stage (∼0.23 ± 0.002). In the GCL, it decreased from P0 to P4 (0.36 ± 0.03), then increased from P4 to P6 (0.48 ± 0.04), and then reduced to the adult stage (0.31 ± 0.16). The INL showed a larger ellipticity than the GCL, especially before P12. As for the spine volume (Figure 6E), the distribution of the two layers was similar except at P2. The mean single spine volume was maximum at P6 (22.86 ± 0.009 μm^3^ for the INL and 30.44 ± 0.01 μm^3^ for the GCL) and in adults (24.29 ± 0.007 μm^3^ for the INL and 24.13 ± 0.016 μm^3^ for the GCL). The total spine volume in the sample area (0.045 mm^2^) of each retina was the same between the two layers at each time point (Supplementary Figure 4), with two peaks at P6 and P35. The total spine number in the two layers increased from P0 to P6 (from 2,058.5 ± 252.08 to 1,8251.5 ± 112.08) and decreased from P6 to the adult stage (6,827.5 ± 412.6; Supplementary Figure 5).

### Proopiomelanocortin-Positive Amacrine Cell Development With Other Retinal Cell Components

**FIGURE 7:**
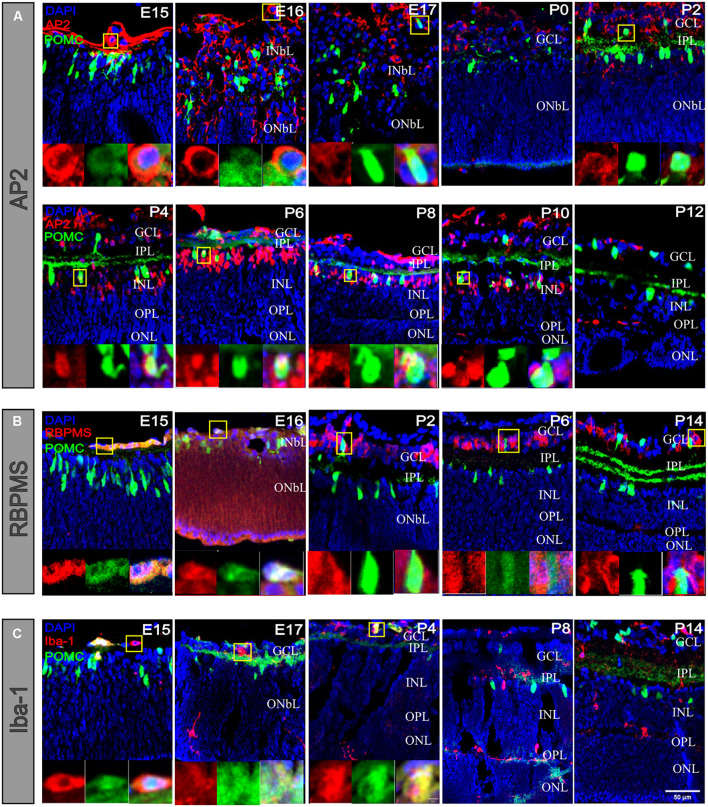
POMC-positive cells costained with RGCs and microglial cells at early developmental stages. **(A)** POMC-positive cell costaining with AP2 (red). There were merged cells at different stages. At the open-eye stage, AP2-positive cells were smaller than POMC-positive cells. Before P2, merged cells were always located on the basal side, whereas after P2, merged cells appeared in the INL. **(B)** POMC-positive cell co-staining with RBPMS (red). There were merged cells at E15 and E16 on the basal side, and in the later stage, there were no prominent merged cells. **(C)** POMC-positive cell costaining with Iba-1 (red). There were also merged cells on the basal side at early stages, such as E15, E17, and P4. With the development of the IPL and OPL, microglia migrated to the outer retina, and no cells merged left. Each image on the bottom is an enlarged image of cells in the yellow boxes. Bar = 50 μm for the large images and 5 μm for the enlarged pictures (*n* = 4 for each time point and each co-staining).

Proopiomelanocortin (POMC) was costained with AP2 (Figure 7A). AP2 is used as a marker for ACs (Zhang et al., 2019). At E15, compared with no costained POMC-positive cells, the AP2-positive costained cells were only located on the basal side margin and showed a more rounded shape. At E16 and E17, the AP2-positive cells were greater in number, and their somas were very clear in the INbL. At P0, the INbL shrank, the IPL appeared, and AP2-positive cells mainly resided in the GCL. At P2, their colocalization was increased. From P4 to P14, AP2 somas become more apparent; among AP2-positive cells and POMC-positive cells, they were about ∼6%–13%, whereas among POMC-positive cells, the percentage of AP2-positive cells was about ∼60%–90%. For all the AP2-positive cells, the POMC-positive cell soma diameters were almost twice that of the POMC-negative cells. There was nearly no costaining when costained with RPBMS (Figure 7B), which used as a pan-RGC maker. At E15 and E16, some cells on the basal side margin showed heavy RPBMS staining, and costaining with POMC was low. At P2, the RPBMS-positive cells were restricted to the GCL and showed a few cells costaining with POMC. At P6 and P14, there was no costaining. Iba-1 is commonly used as a marker for microglia (Figure 7C). At E15 and E17, Iba-1-positive cells were mainly restricted to the basal side, with a few in the INbL. There was little costaining with POMC. At P4, there were also a few costaining cells in the GCL. At P8 and P14, the microglial cells almost resided in the right position and were not costained with POMC.

## Discussion

**FIGURE 8:**
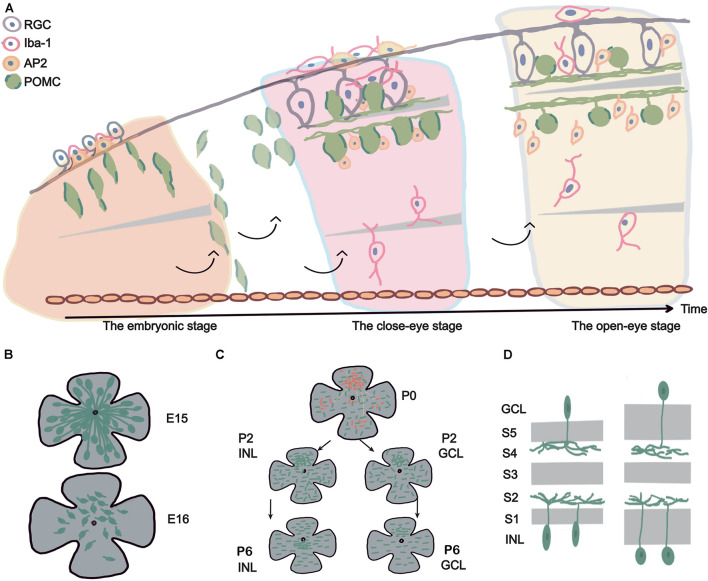
Summary schematic diagram. **(A)** The life span of POMC ACs and related retinal cell development. The figure shows the development of POMC ACs from an embryo to the adult stage (from left to right). The upside is the basal side (RGCs side), and the downside is the apical side (retina pigment cell side). POMC ACs first showed long axons on the basal side, fusiform soma, and had a high density. Then POMC ACs were divided into two layers and formed the inner plexiform layer (IPL). The IPL became wide, two layers of cells increased in number, and the soma became ellipsoidal at P2 to P6. RGCs also developed dendrites to form the IPL. Microglial cells gradually migrated to the OPL. At late developmental stages, POMC ACs had round soma and a stable GCL/INL ratio of about 0.5. **(B)** E15–E16 cliff descent. From E15 to E16, the retina size increased 1.5 times, while the cell density decreased more than 10 times. Cells lost their long axons, and dendrites began to develop. **(C)** Discontinuous development, superior asymmetry, and regularity increased. Considering P2 as an example, the dense pole was in the superior retina, and cells were more developed (shown with red points). These cells were also located in other areas and were separated by less developed cells (black points). The cell mosaic was irregular (with short lines at different directions). At P6, the INL dense pole moved to the optic disc, whereas the GCL dense pole was still in the superior retina. The mosaic regularity reached a peak, with increased cell density. **(D)** Sublamina development. The IPL had five sublamellas; from inner to outer, they are named S1 to S5. The POMC AC dendrites are located in the S2 and S4 lamellas. At later developmental stages, the S2–S4 sublamellas maintained the same width, whereas S1 and S5 widened, and the soma became round.

Our main discovery was that POMC neurons in the retina developed as early as E11 to E13. During development, the cell number, morphology, and distribution of POMC neurons changed. The POMC neuron development can be divided into three stages: the embryonic stage, closed-eye stage, and open-eye stage. During the embryonic stage, there was a dramatic change in the density and distribution of POMC-positive cells at E15-E16. Axon–dendrite trading off and taking turns occurred. During the closed-eye stage, the main difference was the increase in the number of neurons and dendrites, and the position of the density peak was different. Before P6, both the INL and GCL had a density peak in the superior retina. From P6 and onward, the GCL maintained the density peak in the superior retina, while the INL shifted the density peak to around the optic disc. In the open-eye stage, the soma–dendrite distance in the IPL increased, and regularity decreased. A summarizing schematic diagram is shown in Figure 8.

### The Origin of Retinal Proopiomelanocortin Cells

Retinal cells have the same origin, and it is essential to discuss the fate determination and differentiation of these cells. The most accepted model is the multipotent progenitor cell competency and lineage tree differentiation model (Agathocleous and Harris, 2009; Barton and Fendrik, 2015). The progenitors express different transcription factors during specific time windows, which act as intrinsic fate decision clues. Meanwhile, the extrinsic clues such as the environment, location of the progenitor cells, and adjacent cell signals also decide the fate of the cell. Combining both clue types; progenitor cells differentiate in a specific order, that is, ganglion cells, horizontal cells, cone photoreceptors, amacrine cells, and other neurons (Cepko, 2014). Different ACs in different areas of the retina develop at different time points from E12 to P2 (Voinescu et al., 2009). ChAT ACs are the earliest ACs that appear first at about E9–E13.

Interestingly, we found that POMC starts expression at E10 (Supplementary Figure 6); however, it was challenging to decide the precise type of cell-expressing POMC. Notably, at E13, POMC expression was evident in the posterior pole (Figure 1). A previous study reported that at E12.5, RGCs appeared along with the whole INbL layer; however, ACs were restricted to the middle part of RGCs (Trimarchi et al., 2007). In the Hedgehog (Hh) model, fibroblast growth factor (FGF) from the optic stalk, possibly raised by Hh, stimulates the differentiation of RGCs via Ath5. As a result, RGCs produce sonic hedgehog (Shh), which stimulates differentiation in the neighboring retinoblast cells, and thus, a wave of cellular differentiation is produced. The ACs produce their own Shh, which further supports differentiation (Agathocleous and Harris, 2009). That is why at E13, RGCs occupy nearly the full INbL, whereas ACs occupy only the middle part of the INbL. Based on the above evidence, we considered the majority of POMC-positive cells as ACs. We also found some cells at E15 and E16 costained with RBPMS; at E15, E17, and P4, cells were costained with Iba-1. Compared with AP2 costained cells, these cells were only found at the vitreous cavity side at a very low probability. We cannot exclude that some RGCs and microglial cells may also express POMC as well; however, they are also likely multipotential progenitor cells because progenitors can express terminal cell type markers at early embryonic stages (Suzuki et al., 2013), and they existed in the places where RGCs and glial cells arise (Li et al., 2019). From P6 onward, all the POMC-positive cells were POMC ACs.

Inconsistent with a previous report that suggested the number of ACs in the INL keep increasing from E12 to E17 based on immunofluorescent labeling (Hinds and Hinds, 1978), for the first time, we observed the “E15–E16 cliff descent” phenomenon in POMC EGFP mice. The soma numbers peaked with the highest cell density at E15 and decreased dramatically at E16. The retinal area increased approximately 1.5 times (from 1.75 to 2.5 mm^2^; Figure 2); however, the cell density decreased more than 10 times (from 3,253 to 244 cells/mm^2^; Figure 3). This indicated that the decrease in cell density was not due to expansion of the retina. This may indicate that some POMC ACs underwent apoptosis between E15 and E16. Rearrangement of cells through apoptosis and phagocytosis typically occurs in the nervous system or during retinal development (Hinds and Hinds, 1983). It may also be that, at E15, not all POMC neurons were ACs, as revealed by our costaining results (Figure 7), but some may have been POMC RGCs or POMC progenitors. If they were POMC progenitors as we speculated previously, they started transferring into POMC ACs and showed POMC expression, but they lost POMC expression when transforming into other cell types. If they were POMC RGCs, then at E16, the POMC RGCs no longer expressed POMC, and the remaining POMC-expressing cells were POMC ACs. This hypothesis can be supported by the fact that RGCs and ACs share some common markers during early developmental stages (Trimarchi et al., 2007); POMC may be one of them. POMC RGCs themselves may transform into POMC ACs. A previous study reported that some RGCs were differentiated into ACs by losing their axons (Hinds and Hinds, 1983). From our observation, we speculated that, at E15, the POMC neurons were ACs and RGCs because RGCs tend to have stronger and longer basal side axons. A mathematical model suggested that approximately 40% of the ganglion cells lost their axons, which is a large percentage. It also implied that half of the RGCs migrated to the INbL, whereas the other half became displaced ACs (Hinds and Hinds, 1983). The above results are consistent with our findings at E16 (Figures 5, 7A,B). The migration also showed axon–dendrite tradeoffs and taking turns. At E13, most unipolar ACs with a single short axon at the basal side developed after the bipolar ACs (Blume et al., 2020). From E13 to E15, basal axons become longer and stronger, and apical dendrites developed (Figure 5). From E16, the basal axons disappeared. However, further studies are required to investigate the “E15–E16 cliff descent” phenomenon and the POMC neuron identity in the embryonic stage. Still, POMC neurons are guaranteed to be ACs in the adult stage.

### Changes in the Morphology and Distribution of Proopiomelanocortin- Positive Cells Before Eye Opening

From E16 to P14, the distribution of soma number was uneven; especially, there was an asymmetric distribution between the superior and inferior retina. This asymmetric density persisted with the density peak in the superior retina from the embryonic stage to P0 (Figure 2). When cells split into two layers at P2, asymmetric density existed in both layers. From P6, the asymmetric density was restricted in the GCL and disappeared in the INL because the density peak transferred to the optic disc. At the cell level, inhibitory connectivity between starburst cells and ON direction select cells rapidly reorganized to become asymmetric along with the dorso-ventral axis between P6 and P8 (Yonehara et al., 2011). However, it is not known whether the asymmetric connectivity at the cell level is due to the asymmetric distribution or not. This asymmetric distribution was also observed in adult VIP ACs in the mouse retina (Muller et al., 2019). We are the first to report the developmental changes in asymmetric distributions. Both VIP ACs and POMC ACs are ChAT-GABA ACs (Gallagher et al., 2010); however, the AII-AC mosaic is considered to be random and mainly involved in the rod pathway (Keeley and Reese, 2018). The rod distribution in nocturnal rodents is very even (van der Merwe et al., 2018). In mice, it is well-known that S-cones and M-cones are distributed dorsally and ventrally. The majority of the ventral part is made up of S-cones, whereas a small amount of the dorsal part is made up of M-cones (Denman et al., 2018). The dense distribution of ChAT-ACs in the dorsal retina may reveal that ChAT-ACs principally act in the M-cone pathway. The dorsal retina needs more ACs to deal with the sophisticated visual signals, although the RGC spatial distribution is not uneven in adult C57 mice (Salinas-Navarro et al., 2009).

In addition to an asymmetric distribution, the distribution regularities in the dense and spare areas were also changeable (Figure 3). For the same layer, the trends of regularity changes in the dense and sparse areas were the same. However, for the different layers, the trend of regularity changes was different. The regularity kept increasing from E13 to P6 for the INL and then slightly decreased until the adult stage. This regularity peak was consistent with the total spine number peak (Supplementary Figure 4). The association between soma mosaic regularity and dendrite spine numbers was exciting to observe, and it needs more study. For the GCL, the regularity was lowest at P8 and then increased. Overall, the GCL was less regular than the INL. To our knowledge, this is the first report of regularity changes during AC development. A previous study reported the mosaic regularity of cone photoreceptors in the mouse retina from 2.6 to 4.7 at E19–P10 (Fei, 2003). The trend was different, and the cone mosaic was always more regular than the AC mosaic. Maybe it is because cone photoreceptors directly receive light signals; therefore, it needs to be more regular. At the adult stage, the INL regularity index was about 3.0, which is consistent with a previous report of POMC ACs (Gallagher et al., 2010) and similar to AII ACs in the adult rabbit retina (Casini et al., 1995).

Our results also showed that cell development in the dense and sparse areas of the same retina were different (Figure 4). When most of the cells in the dense area have a large dendrite field, a few of the sparse areas have only a tiny dendrite field. The sporadically distributed and less developed ACs in the sparse area were separated by nondeveloped cells, which suggested that the development of cells from dense to sparse areas is not continuous in space. Instead, it is more likely that there was a primary developmental source, and it produced many new small developmental sources. Each small developmental source makes a fully connected dendrite network. The small new developmental source that was selected because of the adjacent RGCs gave an extrinsic developmental clue. This may also support the regularity peak of the AC mosaic at P4. During this developmental period, the mosaic was primarily influenced by the adjacent neurons, and these neurons had higher regularity. From about P8, the uneven development finally disappeared and resulted in asymmetric cell density in the GCL. The asymmetric distribution in the INL disappeared from P6. The reason may be attributed to different functions of the two layers in signal integration. The INL interacts more with bipolar cells (BCs), while the GCL interacts more with RGCs.

Before eye opening, the increase in cell number was a broken line type. There was a slight decrease at P3 and P8 in the INL, whereas in the GCL, only P8 showed a decrease. A previous study has reported that between P3 and P8, many presumptive ACs die in the INL (Young, 1984). After nuclear condensation and pyknosis (apoptosis), these cellular remains of dead cells were phagocytosized by adjacent cells or motile phagocytes. Retina phagocytes, such as microglial cells, usually reside in the OPL (Li et al., 2019), near the INL; this may explain why the cell decrease in the INL was more obvious than in the GCL. The cell number in the GCL was always half compared with the INL at the adult stage (Figures 1, 3). This implies that the INL constitutes 67% of the total POMC AC population (Figure 1), which is less than that of the VIP ACs (98%), as reported by a previous study (Muller et al., 2019). The GCL/INL ratio of soma numbers kept increasing from P0 to P14. Although the cell numbers in both layers kept growing, the GCL cell number increased more quickly. The long-existing GCL density peak pole may serve as a differentiated source.

From P4 to P6, there was soma expansion, which made the soma more ellipsoidal (Figure 6C). Meanwhile, the soma and dendrite positions tended to be more separated. The dendrites gradually contacted each other until there was no spare space (Figure 5), and the two sublaminas of dendrites weaved more regularly to form the IPL. The IPL was roughly divided into ON and OFF laminas and was further divided into five sublaminas. These sublaminas are named S1–S5 from the outer to the inner sides (Sanes and Zipursky, 2010; Olguin et al., 2020). The retina circuit developed via the following stages, including migration, IPL formation, layer formation, sublamina selection, and target selection, resulting in mature neural circuitry. At each stage, different molecular factors regulated this development. Different ACs are located in different sublayers and are connected with different retinal neurons (Reese et al., 2001). ChAT ACs are mainly found in the S2 and S4, whereas VIP ACs are located in S1 and S3–S5 (Hinds and Hinds, 1978) and can be further divided into three subtypes: bistratified INL cells, narrow field INL cells, and GCL cells (Park et al., 2015). Although the two dendrite sublaminas of POMC-ACs were restricted to S2 and S4, there were spines between the two sublaminas (Supplementary Figure 3). This implies that POMC ACs can also be divided into subtypes. During the IPL development, ON and OFF lamellas form first and are divided into 2 to 3 sub-lamellas (Olguin et al., 2020). We found that the lamellas are formed at P2–P4 (Figures 1, 5, 6).

We calculated the total spine volume of each layer and found that they were similar (Supplementary Figure 4). The GCL soma number was half of the INL. Therefore, we speculated that the single-cell dendrite of the GCL was about twice that of the INL, reflecting that the GCL cell fields were twice that of the INL. According to the previous finding, wide-field ACs are derived from ganglion cells by losing the axon and residing in the GCL. In contrast, narrow-field ACs are formed directly from ventricular cells and reside in the INL (Hinds and Hinds, 1978).

### Long-Lasting Development of Proopiomelanocortin-Positive Amacrine Cells After the Eye-Opening Stage

After eye opening, most POMC-ACs do not change both in number and distribution, until the adult stage. We found that the distance between the somas of the GCL and INLs increased slightly. The sublamellas formed at P2–P4, then the distance between S2 and S4 remained unchanged. We also found that the wideness of the IPL was due to the separation of somas and dendrites but not dendritic sublamellas (Figure 6A). The reason may be that the S2–S4 sublamellas were not widened. However, the S1 and S5 sublamellas did widen, and this increase was not due to POMC dendrites but to other retinal neurons, such as parvalbumin ACs (S5) or TH ACs (S1) (Balasubramanian and Gan, 2014). The increased distance between the soma and dendrites seems to be due to the changes of the soma morphology from more ellipsoidal to more round, and the shrunk soma volume transformed into the cell neck. Further studies are recommended to explore the long-lasting IPL sublamellas and cell morphology development.

We believe that subtypes exist for different ACs. This somatotype begins early when the two layers start to develop. For each AC type, the mature cell density was also different. At the adult stage, the mean POMC AC density was about 500 cells/mm^2^ for the INL, which was similar to that of VIP ACs (Muller et al., 2019). Assuming a density of ∼39,700 for ACs/mm^2^ in the INL of the C57BL/6 retina (Jeon et al., 1998), we estimated that POMC ACs in the INL comprise ∼1.3% of the total AC population, which was lower than the POMC AC percentage in AP2-positive cells. In comparison, VIP ACs comprised ∼1.4% (Muller et al., 2019), TH ACs only comprised ∼0.12% (Munteanu et al., 2018), AII ACs comprised ∼11.2%, and vGluT3 ACs (a kind of glycinergic AC) comprised ∼2.0% (Keeley et al., 2014). From this data, we believe that POMC ACs are also important, like VIP-ACs.

In summary, the development of POMC ACs from embryonic stages to adulthood stage displayed an exciting trilogy, and each stage has specific events. This developmental pattern can reference other ACs or even retinal neuron development based on comprehensive and detailed observations. As POMC ACs secrete neuropeptides that have potentially massive effects on visual physiology, knowing the full lifecycle, of POMC neurons will be easy for clinicians and researchers to understand and can then develop treatments for various eye diseases.

## Data Availability Statement

The original contributions presented in the study are included in the article/Supplementary Material, further inquiries can be directed to the corresponding authors.

## Ethics Statement

The animal study was reviewed and approved by the Tab of Animal Experimental Ethical Inspection of the First Affiliated Hospital, School of Medicine, Zhejiang University (Approval No. 2021001).

## Author Contributions

YZ, YS, and JF designed the research and edited the manuscript. XZ, XW, WP, and SW performed the research. XZ, XW, and RU analyzed the data. XZ wrote the first draft of the manuscript. RU wrote the manuscript. All authors contributed to the article and approved the submitted version.

## Conflict of Interest

The authors declare that the research was conducted in the absence of any commercial or financial relationships that could be construed as a potential conflict of interest.

## Publisher’s Note

All claims expressed in this article are solely those of the authors and do not necessarily represent those of their affiliated organizations, or those of the publisher, the editors and the reviewers. Any product that may be evaluated in this article, or claim that may be made by its manufacturer, is not guaranteed or endorsed by the publisher.

## Supplementary Material

Supplementary Figure 1 Retina whole-mount view showing E13 to the adult stage. The coordinate axis indicates the actual retina size. From E13 to the adult stage, the retina whole-mount size became larger. Each retina was seated with the superior upside and nasal at the right side. The scale bar represents the POMC-positive cell density. Blue represents the denser area, and yellow represents the sparse area. The black line shows the mean density contour line. E13 to P0 had only one layer, whereas P2 to adult had two layers. The dense pole was always located in the superior retina from E13 to P6. However, from P6, the INL dense pole transferred to the optic disc, the GCL pole was still in the superior retina.

Supplementary Figure 2 The nearest nearby distance frequency distribution histogram. This figure represent the dense and sparse areas of each layer. The Y-axis shows the frequency and the X-axis shows the NND (×10 μm).

Supplementary Figure 3 Small spines between the two dendrite sub-lamellas. This figure shows the weaving of the two layers of dendrites and provides evidence for the bitrate subtype of POMC-ACs. Bar = 20 μm.

Supplementary Figure 4 The changes in the total spine volume of each layer. The total spine volume in a 0.045 mm^2^ sample area was the same between the two layers; there were two peaks; that is, at P6 and P35 (*n* = 4 for each timepoint).

Supplementary Figure 5 The change in the total spine number of the two layers. The total spine number was masked by Imaris spots function. From P0 to P6, the spine number in a 0.045 mm^2^ sample area increased and then decreased (*n* = 3 for each timepoint).

Supplementary Figure 6 The E10 section showed little POMC expression.

## Abbreviations

POMC-AC: Proopiomelanocortin-positive amacrine cells
RGC: Retina ganglion cell
GCL: Ganglion cell layer
INL: Inner nuclera layer
ONL: Outer nuclera layer
INbL: Inner neuroblastic layer
ONbL: Outer neuroblastic layer
IPL: Inner plexiform layer
OPL: Outer plexiform layer
D: Dorsal
V: Ventral
I: Inperior
S: Superior
T: Temporal
N: Nasal
VIP: vasoactive intestinal peptide
ChAT: choline acetyltransferase
TH: tyrosine hydroxylase
MSH: melanocyte-stimulating hormones a
ACTH: adrenocorticotropic hormone
NND: nearest neighbor distances.
