# Supplementary figures and images for: Trilogy Development of Proopiomelanocortin Neurons From Embryonic to Adult Stages in the Mice Retina
Source: Front Cell Dev Biol. 2021 Oct 5;9:718851. doi: 10.3389/fcell.2021.718851 (PMC8523858; doi:10.3389/fcell.2021.718851)

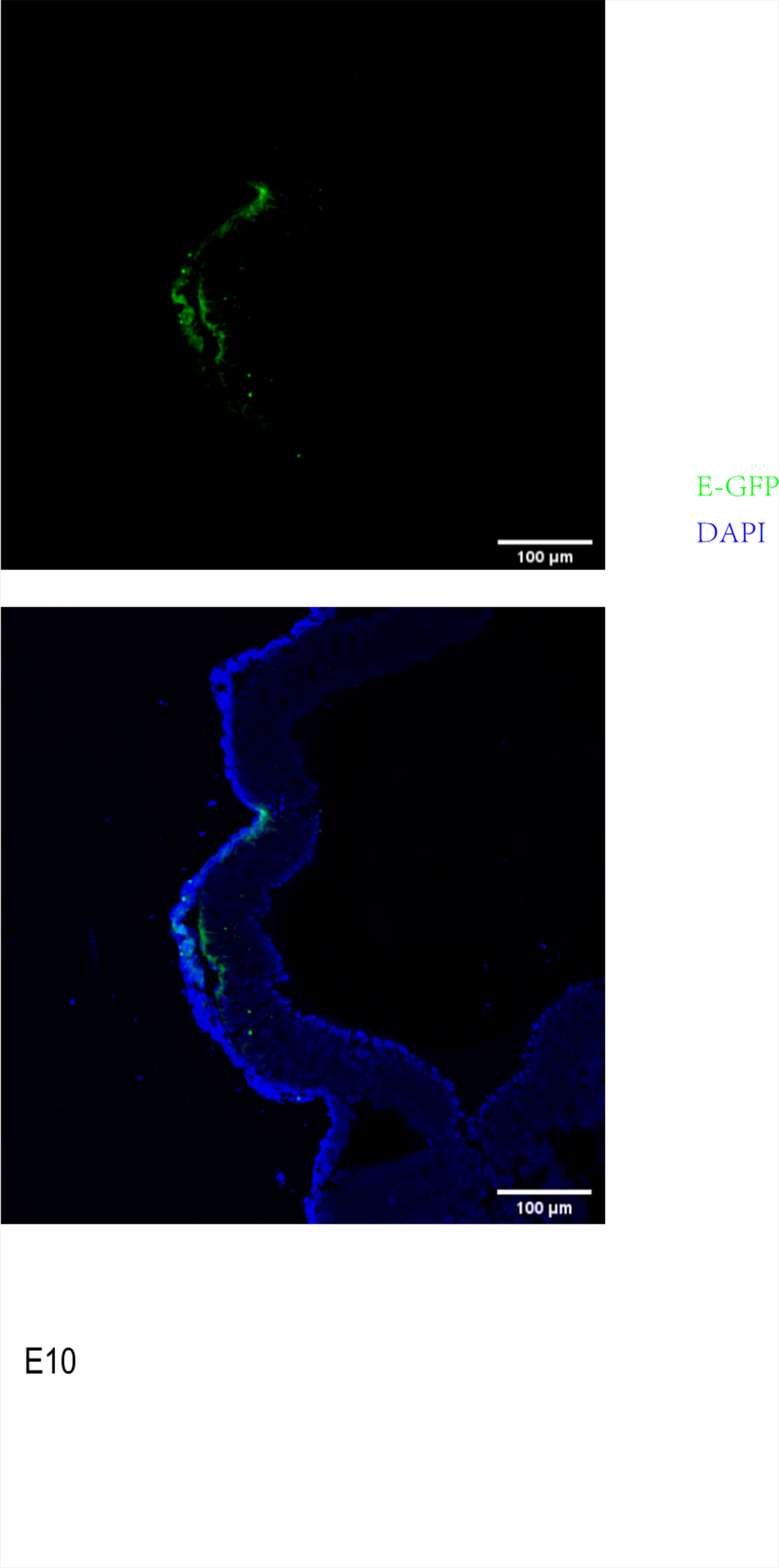

Supplement: Supplementary file 1 [file Image_7.TIFF]

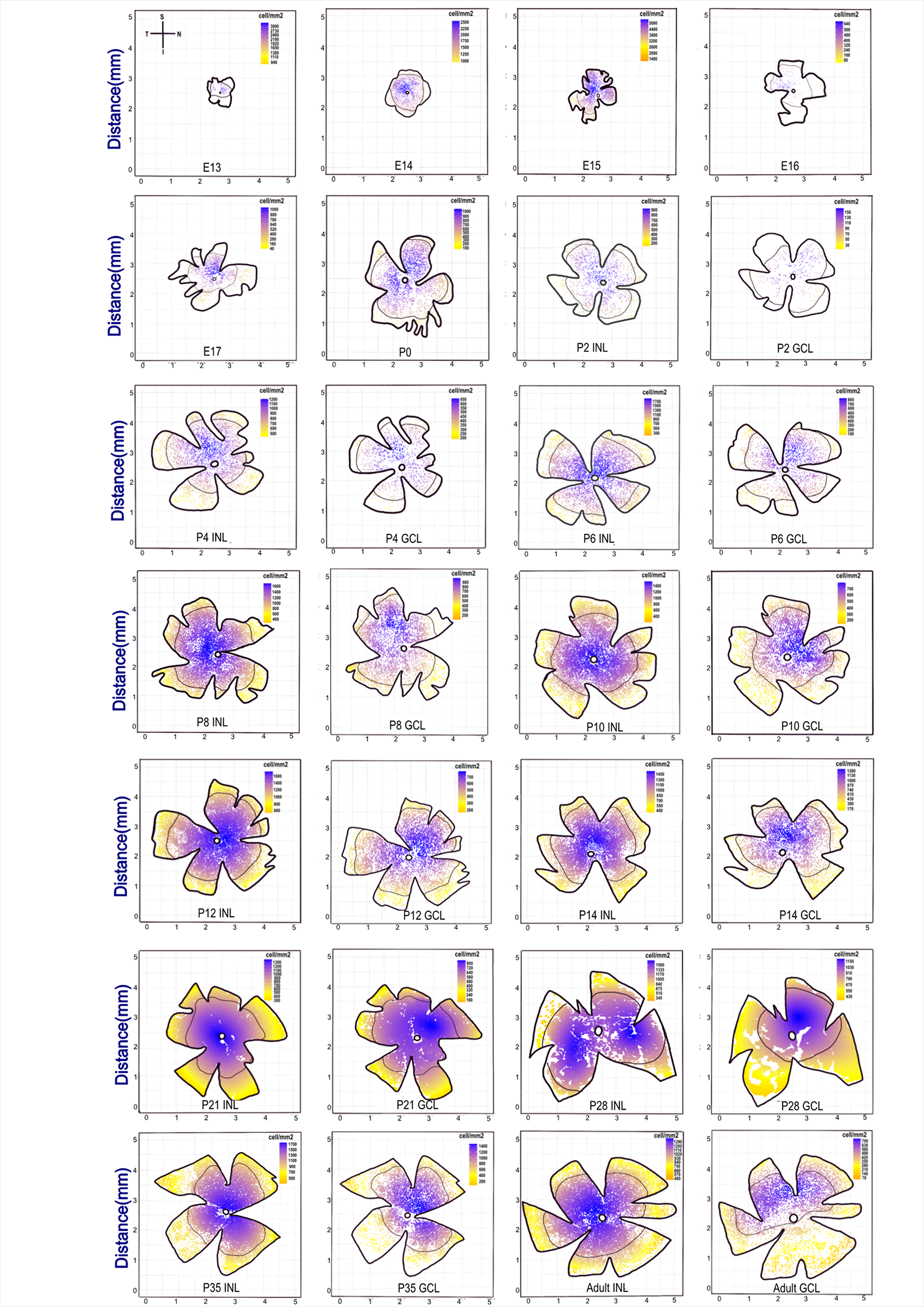

Supplement: Supplementary Figure 1 — Retina whole-mount view showing E13 to the adult stage. The coordinate axis indicates the actual retina size. From E13 to the adult stage, the retina whole-mount size became larger. Each retina was seated with the superior upside and nasal at the right side. The scale bar represents the POMC-positive cell density. Blue represents the denser area, and yellow represents the sparse area. The black line shows the mean density contour line. E13 to P0 had only one layer, whereas P2 to adult had two layers. The dense pole was always located in the superior retina from E13 to P6. However, from P6, the INL dense pole transferred to the optic disc, the GCL pole was still in the superior retina. [file Image_1.TIFF]

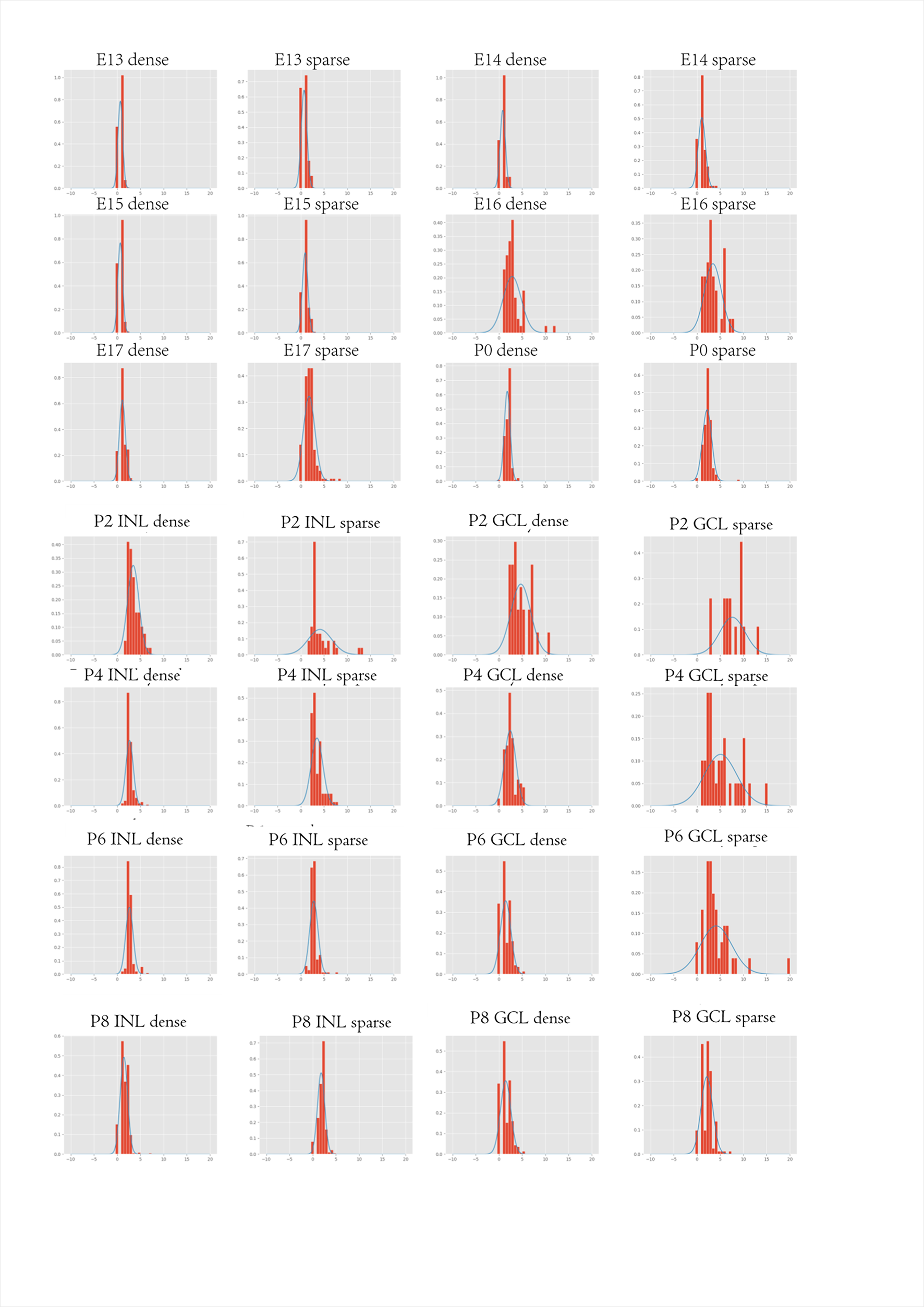

Supplement: Supplementary Figure 2 — The nearest nearby distance frequency distribution histogram. This figure represent the dense and sparse areas of each layer. The Y-axis shows the frequency and the X-axis shows the NND (×10 μm). [file Image_2.TIFF]

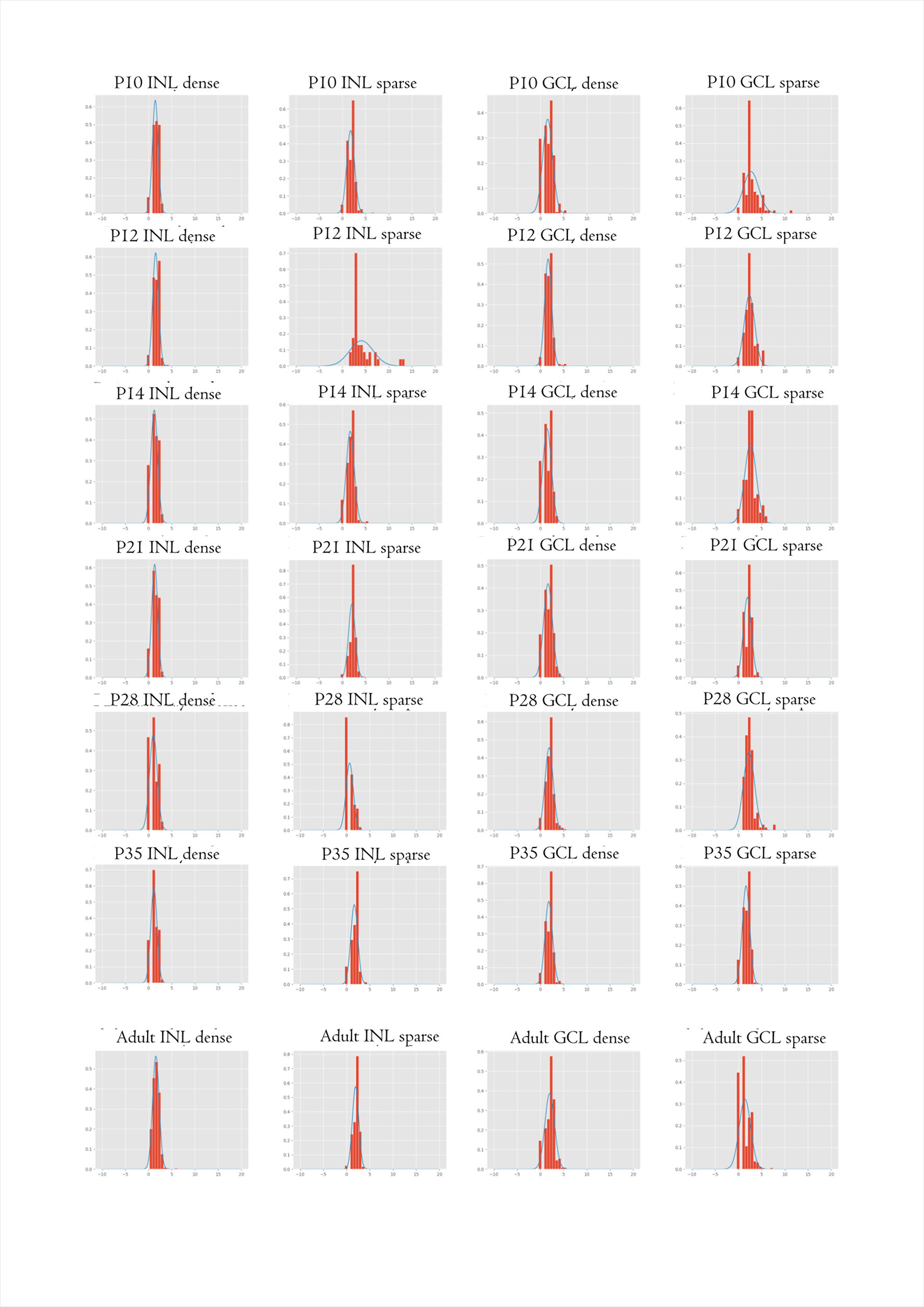

Supplement: Supplementary Figure 3 — Small spines between the two dendrite sub-lamellas. This figure shows the weaving of the two layers of dendrites and provides evidence for the bitrate subtype of POMC-ACs. Bar = 20 μm. [file Image_3.TIFF]

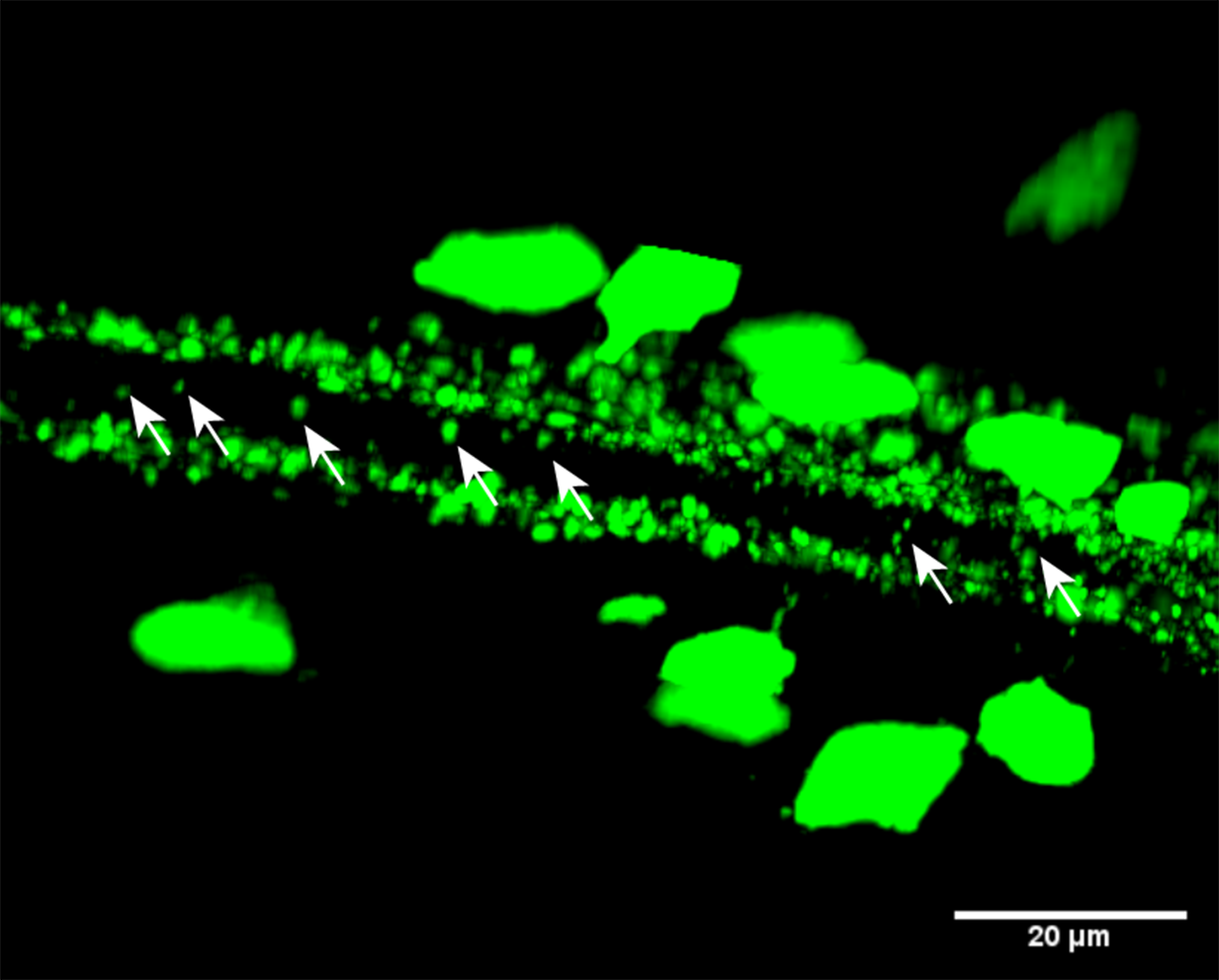

Supplement: Supplementary Figure 4 — The changes in the total spine volume of each layer. The total spine volume in a 0.045 mm2 sample area was the same between the two layers; there were two peaks; that is, at P6 and P35 (n = 4 for each timepoint). [file Image_4.TIFF]

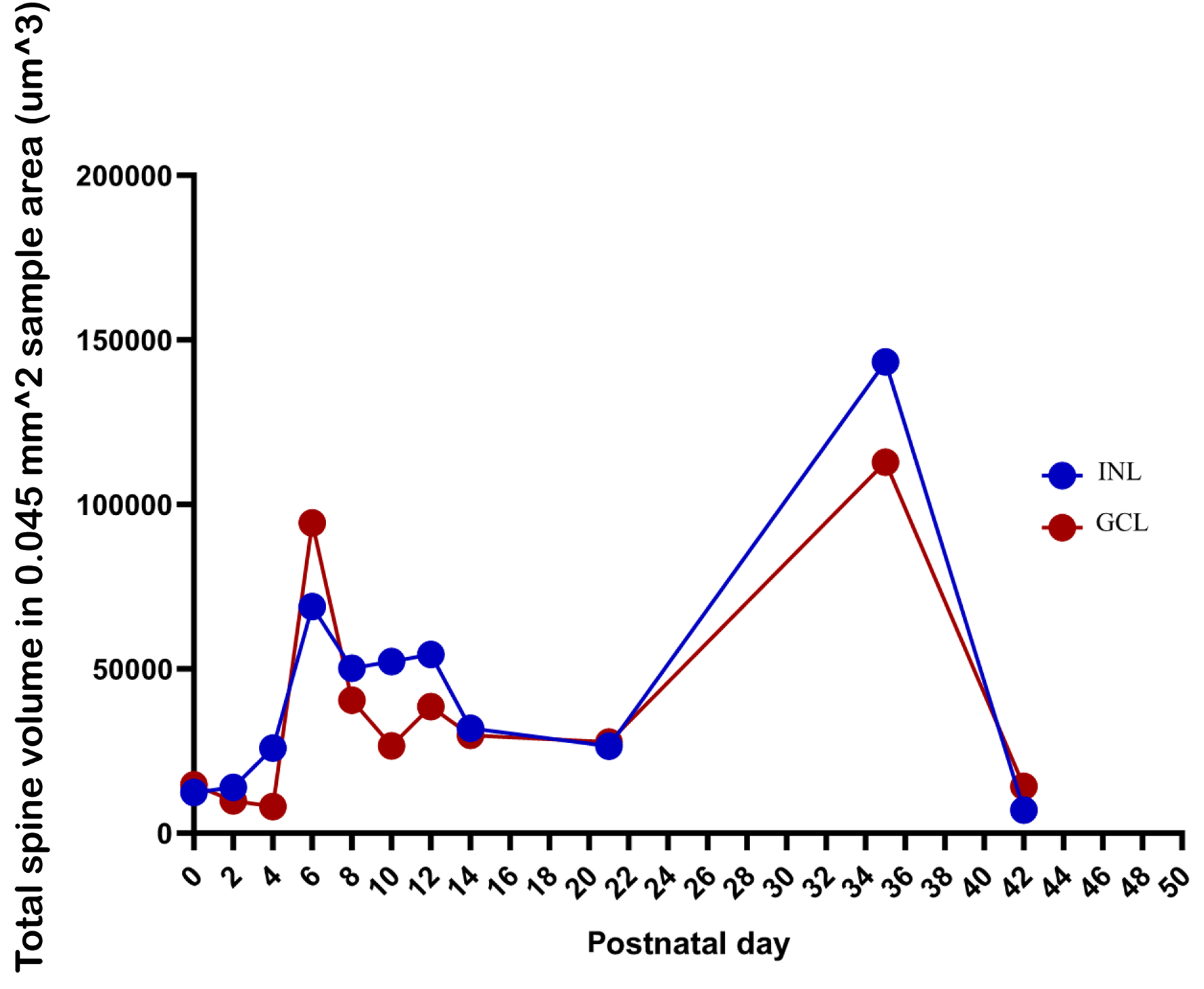

Supplement: Supplementary Figure 5 — The change in the total spine number of the two layers. The total spine number was masked by Imaris spots function. From P0 to P6, the spine number in a 0.045 mm2 sample area increased and then decreased (n = 3 for each timepoint). [file Image_5.TIF]

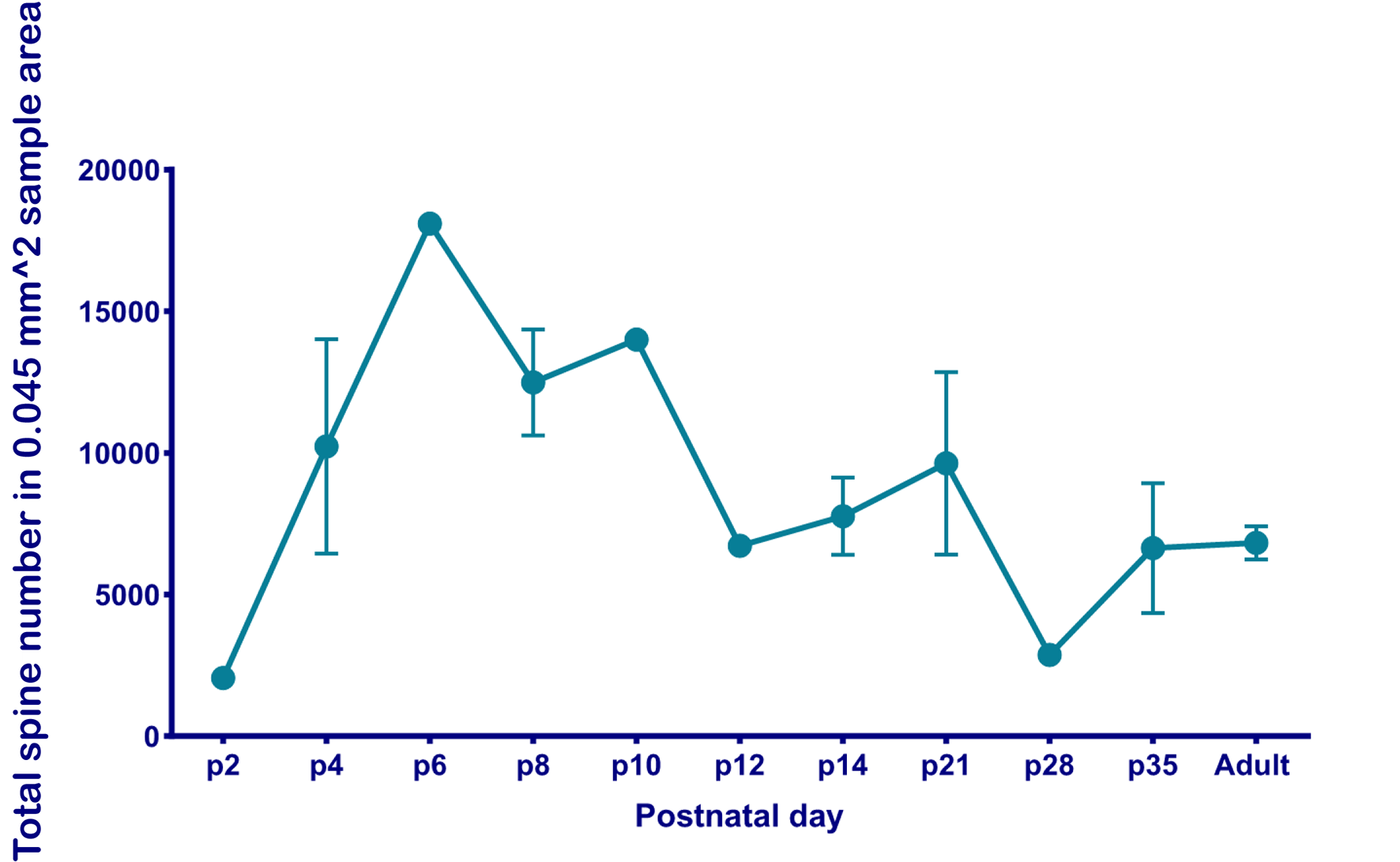

Supplement: Supplementary Figure 6 — The E10 section showed little POMC expression. [file Image_6.TIF]
